# Supplementary material for: Pretreatment of Glioblastoma with Bortezomib Potentiates Natural Killer Cell Cytotoxicity through TRAIL/DR5 Mediated Apoptosis and Prolongs Animal Survival
Source: Cancers (Basel). 2019 Jul 17;11(7):996. doi: 10.3390/cancers11070996 (PMC6678126; doi:10.3390/cancers11070996)
Supplement: Supplementary file 1 [file cancers-11-00996-s001.pdf]

Supplementary Materials: Pretreatment of Glioblastoma with Bortezomib Potentiates Natural Killer Cell Cytotoxicity through TRAIL/DR5 Mediated Apoptosis and Prolongs Animal Survival

Andrea Gras Navarro, Heidi Espedal, Justin Vareecal Joseph, Laura Trachsel-Moncho, Marzieh Bahador, Bjørn Tore Gjertsen, Einar Klæboe Kristoffersen, Anne Simonsen, Hrvoje Miletic, Per Øyvind Enger, Mohummad Aminur Rahman and Martha Chekenya

1. Supplementary Methods

1.1. Cell Line Authentication by STR Profiling

Authentication of cell line identity was done using highly-polymorphic short tandem repeat loci (STR) analysis against the original patient tumour DNA as template (Microsynth, Balgach, Switzerland). Briefly, approximately 30 mg tissue was isolated from cryosectioned, frozen tumour biopsies (2012-018, BG7, BG8 and BG9) and from corresponding tumour lines (2012-018, BG7, BG8, BG9 and P3). DNA was extracted using DNeasy Blood & Tissue Kits (Qiagen, Hilden, Germany; Cat No./ID: 69506). 50 µL (50–150 ng/µL) of gDNA was delivered to Microsynth for DNA fingerprinting.

1.2. Western Blotting

Cells were lysed in Kinexus lysis buffer: 20 mM MOPS, 5 mM EDTA, 2 mM EGTA, 30 mM NaF, 0.5% Triton X, 1 mM PMSF, pH 7.2, protease inhibitor (cocktail tablet, Roche; Basel, Switzerland), and phosphatase inhibitor (cocktail tablet, Roche). Samples (20 µg) were run on SDS/PAGE with NuPage precast 4–12% gradient gels (Invitrogen; Carlsbad, CA, USA) blots were incubated overnight at 4 °C with primary antibodies (Table S1) followed by incubation for 1.5 h at RT with a species specific secondary HRP-conjugated antibody diluted 1:10,000. Chemiluminescence detection was performed with Super Signal West Femto Maximum Sensitivity Substrate (Thermo Fisher Scientific; Waltham, MA, USA) on the LAS-3000 (Fujifilm Medical Systems Inc.; Stamford, CT, USA). Relative protein expression levels were normalized to GAPDH and quantified using Image J software (NIH; Bethesda, MD, USA).

1.3. Immunohistochemistry

Formalin-fixed paraffin-embedded (FFPE) sections of brains were subjected to IHC staining using standard procedures and stained with antibodies indicated in Table S1. The Ki67 labeling index and CD31 positive microvascular density were quantified in 5 microscopic high-power fields (400× magnification) in all animals in each study group as previously described [1].

1.4. Magnetic Resonance Imaging

MRI was performed with the 7 Tesla Bruker Pharmascan system (Bruker Corporation; Ettlingen, Germany) and linear volume RF coil internal diameter 23 mm. T1-weighted MR imaging was obtained using a rapid acquisition relaxation enhancement (RARE) sequence with the following parameters: RT = 900.0 ms; TE = 9.00 ms; rare factor = 4, a slice thickness of 1.0 mm and resolution at 0.0078 cm/pixel. T2-weighted MR imaging was obtained using a RARE sequence with the following parameters: RT = 3500.0 ms; TE = 35.22 ms; rare factor = 8, a slice thickness of 1.0 mm and resolution at 0.0078 cm/pixel. MRI scanning was performed 2–3 weeks post tumour implantation to establish contrast enhancement defining tumor take, and thereafter 4 animals from each treatment group were longitudinally scanned at days 40, 49, 56 and 66 after spheroid implantation. Tumour volumes were calculated in Osirix v 8.5, and each measurement involved (1) T2-weighted scan and (2) post-contrast

T1-weighted scan with 0.1 mL of Dotarem®. Tumour volume doubling time was calculated using the following equation:

$$Doubling\ time\ (days) = \frac{duration\ (days) * \log(2)}{\log(final\ tumor\ volume) - \log(intital\ tumor\ volume)}$$

Table S1. List of antibodies used in flow cytometry phenotyping, divided by experiment frames, in WB and IHC.

Frame	Antibodies	Fluorochrome	Cat. No.	Company
**Apoptosis**	Annexin V	Pacific Blue™	640928	Biolegend (Oslo, Norway)
	PI	PI	640928	Biolegend
**Autophagy Flux**	p62/SQSTM1	Alexa Fluor® 647	M162-A64	MBL (Des Plaines, IL, USA)
	LC3A + LC3B	DyLight 488	PA5-22731	Invitrogen (Hämeenlinna, Finland)
	Live/Dead Fixable Near-IR	APC-Cy7	L10119	Invitrogen
**GBM cell markers**	ULBP-1	*	MAB1380	R&D System (Abingdon, UK)
	ULBP-3	*	MAB1517	R&D System
	MICA	APC	FAB1300A	R&D System
	MICB	PE	FAB1599P	R&D System
	TRAIL-R2/TNFRSF10B	Alexa Fluor® 700	FAB6311N	R&D System
	CD112 (nestin-2)	PerCP-Cy5.5	337416	Biolegend
	GFAP	FITC	25-0319	eBioscience (Ireland, UK)
	nestin	PerCP-Cy5.5	561331	BD Bioscience (Trondheim, Norway)
	CD45	V500	560779	BD Bioscience
	HLA-ABC	APC	562006	BD Bioscience
	HLA-DR, DQ, DP	FITC	562008	BD Bioscience
	F(ab')2-anti-IgG	* Pacific Orange	P31585	Invitrogen
	Live/Dead Fixable Near-IR	APC-Cy7	L10119	Invitrogen
**NK cell markers**	CD56	V450	560360	BD Bioscience
	CD3	V500	560770	BD Bioscience
	CD16	FITC	555406	BD Bioscience
	CD69	PE	555531	BD Bioscience
	NKG2D	PE-Cy7	562365	BD Bioscience
	NKp46	APC	558051	BD Bioscience
	NKG2A	Alexa Fluor® 700	FAB1059N	R&D System
	NKG2C	Alexa Fluor® 488	FAB138G	R&D System
	CD57	PE-Cy5	Ab25445	Abcam (Cambridge, UK)
	KIR2DL4	APC	FAB2238A	R&D System
	KIR2DL1	Alexa Fluor® 700	FAB1844N	R&D System
	KIR2DL3	APC	FAB2014A	R&D System
	KIR2DS4	PE	MAB1847	R&D System
	KIR3DL1	Alexa Fluor® 700	312712	Biolegend
	KIR2DL2/3	PE	13-092-618	Miltenyi Biotec (Gladbach, Germany)
	KIR3DL1/2	PE	130-095-205	Miltenyi Biotec
	KIR2DS2	¤	ab128356	Abcam
	F(ab')2-anti-IgG	¤ Qdot585	Q-11411MP	Invitrogen
	Live/Dead Fixable Near-IR	APC-Cy7	L10119	Invitrogen
**WB**	PARP	-	9542S	Cell Signaling Technology (Danvers, MA, USA)
	Cleaved PARP	-	5625S	Cell Signaling Technology
	Caspase 3	-	9662S	Cell Signaling Technology
	Cleaved caspase 3	-	9664S	Cell Signaling Technology
	SQSTM1/p62	-	5114S	Cell Signaling Technology
	LC3A/B	-	12741S	Cell Signaling Technology
	GAPDH	-	5174S	Cell Signaling Technology
**IHC**	CD31 (Pecam-1)	^	DIA-310	Dianova (Hamburg, Germany)
	Rat IgG	^ Biotinylated	BA-4001	Vector Laboratories (Burlingame, CA, USA)
	nestin	#	MAB5326	Merck Life Science (Oslo, Norway)
	Ki67	#	M7240	Dako (Carpinteria, CA, USA)
	Mouse IgG	# HRP	K400711-2	Dako

*, ¤, ^, #: unconjugated primary antibodies that were stained with the secondary antibody marked with the same sign.

Table S2. IC50s of BTZ in monotherapy and BTZ + NK combination therapy for P3, 2012-018, BG7 and NHA.

Cell line	Therapy	Bortezomib IC50 (nM)			
		BTZ treatment: 24 h		BTZ treatment: 48 h	
		Flow	Clonogenic	Flow	Clonogenic
P3	BTZ Monotherapy	959 ± 208 ***	12.8 ± 0.9 *	18.3 ± 3.3	11 ± 1 *
	BTZ + NK cells Combination	26 ± 8 ***	9.5 ± 1 *	17 ± 5	8 ± 1 *
2012-018	BTZ Monotherapy	1343.7 ± 197.2 ****	9.7 ± 0.9 ***	16.3 ± 3.8	6.8 ± 0.4 **
	BTZ + NK cells Combination	63 ± 35.3 ****	4.3 ± 0.5 ***	10 ± 1	3.5 ± 0.2 **
BG7	BTZ Monotherapy	12.1 ± 0.7	7.5 ± 0.3	4 ± 0.3	5.4 ± 0.5
	BTZ + NK cells Combination	9 ± 0.17	6 ± 0.4	2.5 ± 0.9	3.7 ± 0.1
NHA	BTZ Monotherapy	1282.2 ± 242.5 ****	N/A	36.5 ± 4.5	N/A
	BTZ + NK cells Combination	39.4 ± 6.2 ****	N/A	28.2 ± 5.6	N/A

Comparisons between BTZ monotherapy and BTZ + NK cells combination therapies. Two-way ANOVA, Bonferroni's multiple test, * $p < 0.05$, ** $p < 0.01$, *** $p < 0.001$ and **** $p < 0.0001$.

2. Results

To understand the basis of the killing efficacy, we genotyped the donor NK and GBM cells for KIR receptor and HLA ligands, respectively. Five allogeneic donors possessed NK cells with KIR receptor-ligand mismatch (Table S3, orange mark), where their licenced NK cells' KIR receptors were not compatible with the GBM patients' class I HLA ligands. In this setting, no inhibitory signals are transmitted to the KIR receptor, resulting in predominance of activation signals and enhanced NK cell mediated killing of target cells (Table S3, Figure 1B). However, three of these donors possessed licenced inhibitory KIR2DL1 and KIR3DL2 receptors that encountered inhibitory signals from cognate class I HLA ligands in the 2012-018 GBM cells (Table S3, blue mark), thus potentially dampening their overall cytolytic activity (Figure 1B). All 3-patient derived tumours had cognate HLA-ligands that provided strongly inhibitory signals to licensed autologous NK cells' inhibitory KIR receptors (Table S4). The BG9 patient's NK cells possessed inhibitory KIR2DL2 and KIR2DL3 educated within the cognate ligand HLA-C1 *07:02 (Table S4). This would render these NK cells inhibitory to self-cells. BG8 and BG7 tumours that were responsive to autologous NK cells did not possess HLA-C1 *07:02 epitope. As this was a distinguishing feature of BG9 tumour, this might be the reason for the resistance to autologous NK cells. The resistance could also be explained by low basal NKG2D ligands expression and/or inability of BTZ to enhance their expression as we observed in 2012-018. Unfortunately, stress ligand expression in BG9 cells could not be assessed by flow cytometry due to unavailability of the cells for large scale analysis.Taken together, both allogeneic and autologous NK cells variably killed GBM cells, where potency was modulated by licenced NK cells inhibitory KIR and activating receptor-ligand interactions.

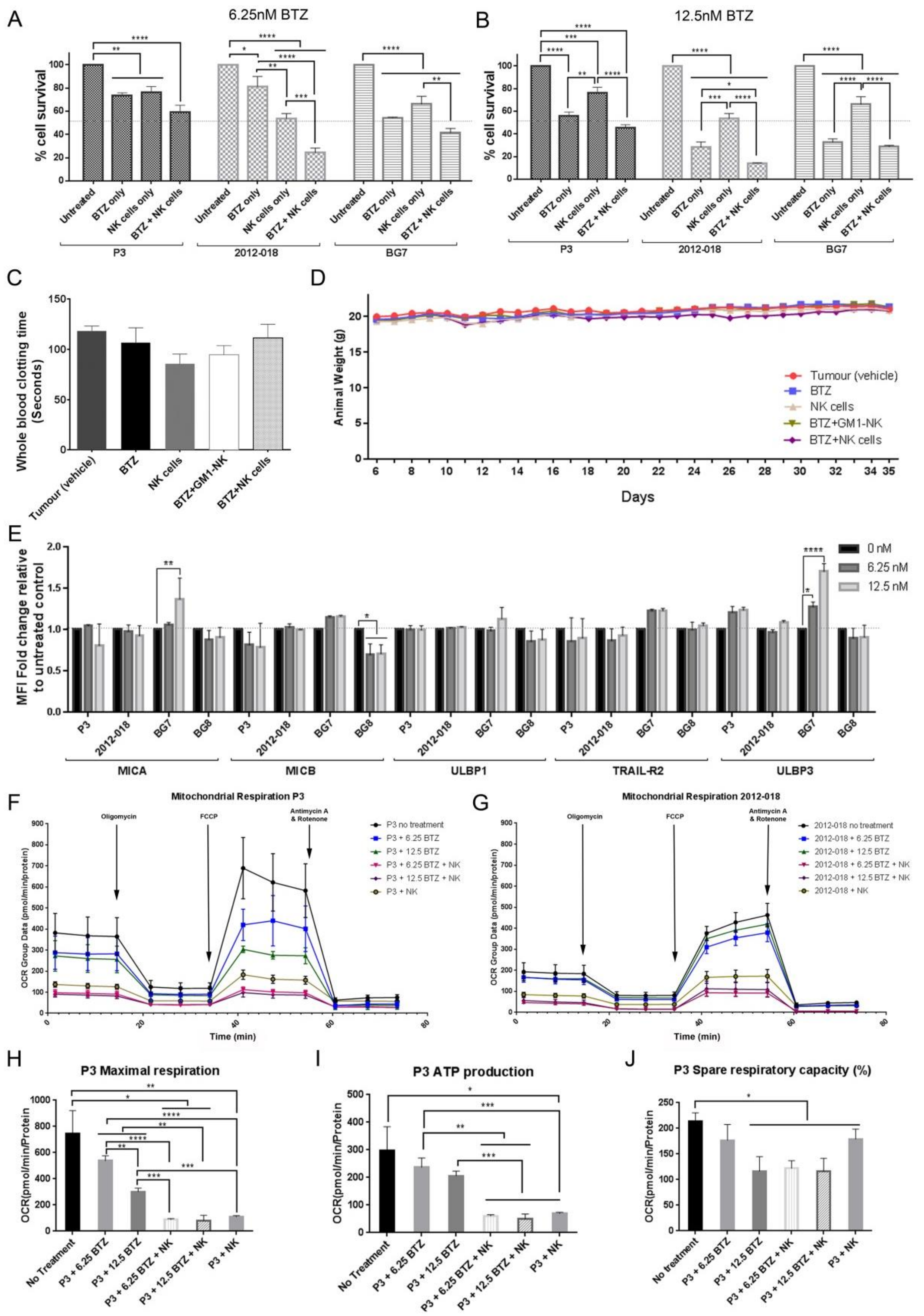

Figure S1. *Cont.*

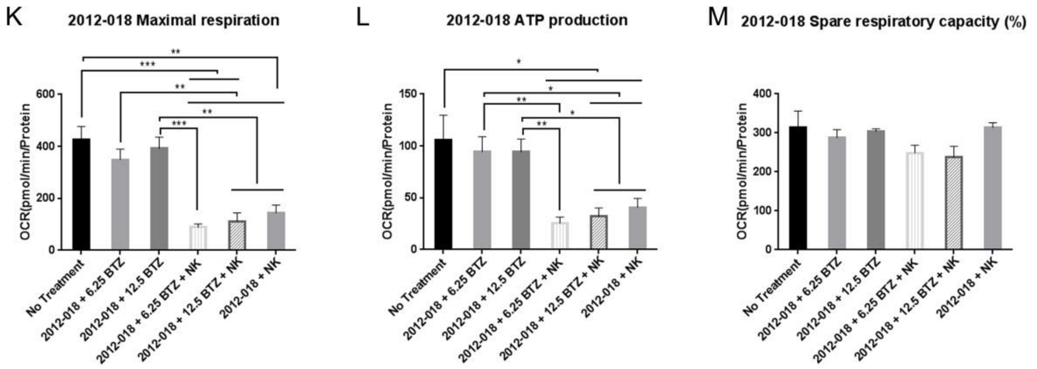

Figure S1. Combination bortezomib + NK cells treatment is more effective against GBM than bortezomib and NK cell monotherapy. P3, 2012-018 and BG7 cells in clonogenic assays untreated, treated with BTZ, with 5:1 of NK cells and with combination of BTZ + 5:1 NK cells; treated with allogeneic NK cells and (**A**) 6.25 nM or (**B**) 12.5 nM BTZ. (**C**) Whole blood clotting time (sec) of animals from the treatment groups indicated, and (**D**) animal body weight. (**E**) % fold change relative to (black) untreated control cells P3, 2012-018, BG7 and BG8 of NKG2D ligands: MICA, MICB, ULBP1, ULBP3, and the receptor TRAIL-R2 after treatment with 6.25 nM (dark grey) and 12.5 nM (light grey) BTZ. Profile of mitochondrial respiration based on oxygen consumption rate (OCR) obtained with the Seahorse assay for (**F**) P3 and (**G**) 2012-018 GBM cells with the different treatment conditions: GBM untreated, GBM treated with 6.25 nM or 12.5 nM BTZ, GBM treated with combination of 6.25 nM or 12.5 nM BTZ + 5:1 allogeneic NK cells, and GBM treated with 5:1 allogeneic NK cells. P3 cell measurements of (**H**) maximal respiration, (**I**) ATP production and (**J**) spare respiratory capacity after the different treatments. 2012-018 cell measurements of (**K**) maximal respiration, (**L**) ATP production and (**M**) spare respiratory capacity after the different treatments. Data represents the mean ± SEM of $n = 3$ independent experiments. Two-way ANOVA, Bonferroni's multiple comparison test, * $p < 0.05$; ** $p < 0.01$; *** $p < 0.001$; **** $p < 0.0001$.

Table S3. KIR-HLA genotype for P3, 2012-018 and BG7 with healthy donors used for cytotoxicity assays.

Receptor Type	KIR Receptor	Epitope Group	HLA Ligand	Tumor	Donors			Tumor	Donors			Tumor	Donors			KIR Receptor
				p3	53	54	55	2012-018	57	60	65	BG7	75	77*	36	
Inhibitors	KIR2DL1	C2	C*0401	-	+	+	+	+	+	+	+	-	+	+	-	KIR2DL1
			C*0501	+	+	+	+	-	+	+	+	-	+	+	-	
	KIR2DL2	C1	C*0102	+	-	+	+	-	-	+	-	-	+	-	+	KIR2DL2
			C*0701	-	-	+	+	+	-	+	-	+	+	-	+	
			C*0304	-	-	+	+	-	-	+	-	+	+	-	+	
			C*0501**	+	-	+	+	-	-	+	-	-	+	-	+	
	KIR2DL3	C1	C*0102	+	+	+	+	-	+	-	+	-	+	+	-	KIR2DL3
			C*0701	-	+	+	+	+	+	-	+	+	+	+	-	
			C*0304	-	+	+	+	-	+	-	+	+	+	+	-	
			C*0501**	+	+	+	+	-	+	-	+	-	+	+	-	
	KIR3DL1	Bw4	B*2705	+	+	+	+	-	+	+	+	-	+	+	+	KIR3DL1
			B*4402	+	+	+	+	-	+	+	+	-	+	+	+	
			B*3701	-	+	+	+	+	+	+	+	-	+	+	+	
	KIR3DL2	A	A*0301	-	+	+	+	+	+	+	+	-	+	+	+	KIR3DL2
			A*1101	-	+	+	+	-	+	+	+	+	+	+	+	
	KIR3DL3		Unknown		+	+	+		+	+	+		+	+	+	KIR3DL3
	KIR2DL5		Unknown		-	+	-		-	+	-		+	-	-	KIR2DL5
	KIR2DL4		HLA-G	-	+	+	+	-	+	+	+	+	+	+		KIR2DL4
Activators	KIR2DS1	C2	C*0401	-	-	-	-	+	-	+	-	-	+	-	-	KIR2DS1
			C*0501	+	-	-	-	-	-	+	-	-	+	-	-	
	KIR2DS2		unknown		-	+	+		-	+	-		+	-	+	KIR2DS2
	KIR2DS3	C1	C*0102	+	-	+	-	-	-	-	-	-	+	-	-	KIR2DS3
			C*0701	-	-	+	-	+	-	-	-	+	+	-	-	
			C*0304	-	-	+	-	-	-	-	-	+	+	-	-	
	KIR2DS4	C2	C*0401	-	+	+	+	+	+	+	+del	-	+del	+	+del	KIR2DS4
			C*0501	+	+	+	+	-	+	+	+del	-	+del	+	+del	
	KIR2DS5		unknown		-	-	-		-	+	-		+	-	-	KIR2DS5
	KIR3DS1		unknown		-	+	-		-	+	-		+	-	-	KIR3DS1

For NK cells: - and green, NK cells are negative for KIR; + and red, NK cells are positive for KIR; +del and red, NK cells have partial deletion KIR2DS4; + and blue, NK cells have been educated in the context of the cognate HLA, which is present in the GBM; and + and orange, NK have been educated in the context of the cognate HLA, which is missing in the GBM, indicating a potential functional mismatch of inhibitory KIRs. For GBM: +, GBM are positive for the specific HLA ligand; -, GBM are negative for the HLA ligand. Red, GBM are positive for at least one of the HLA ligands

of the cognate KIR; green, GBM are negative for all HLA ligands of the cognate KIR; and white, HLA ligand is unknown. * Donor 77 HLA-typing not available. ** C2 epitope recognized by KIR2DL2/3.

Table S4. KIR-HLA genotype for BG7, BG8 and BG9 with autologous NK cells used for cytotoxicity assays.

Receptor Type	KIR Receptor	Epitope Group	HLA Ligand	Tumor BG7	BG7-PBMC	Tumor BG8	BG8-PBMC	Tumor BG9	BG9-PBMC	KIR Receptor
Inhibitors	**KIR2DL1**	**C2**	C*0401	-	+	+	+	-	+	**KIR2DL1**
	**KIR2DL2**	**C1**	C*0701	-	-	+	+	-	+	**KIR2DL2**
			C*0304	+	-	-	+	-	+	
			C*0702	-	-	-	+	+	+	
	**KIR2DL3**	**C1**	C*0701	-	+	+	+	-	+	**KIR2DL3**
			C*0304	+	+	-	+	-	+	
			C*0702	-	+	-	+	+	+	
	**KIR3DL1**	**Bw4**	B*2705	-	+	-	+	-	+	**KIR3DL1**
			B*4402	-	+	-	+	-	+	
			B*3701	-	+	-	+	-	+	
	**KIR3DL2**	**A**	A*0301	-	+	+	+	-	+	**KIR3DL2**
			A*1101	+	+	-	+	-	+	
	**KIR3DL3**		Unknown		+		+		+	**KIR3DL3**
	**KIR2DL5**		Unknown		+		-		-	**KIR2DL5**
	**KIR2DL4**		HLA-G	+	+		+		+	**KIR2DL4**
Activators	**KIR2DS1**	**C2**	C*0401	-	+	-	-	-	-	**KIR2DS1**
			C*0501	-	+	-	-	-	-	
	**KIR2DS2**		unknown		-		+		+	**KIR2DS2**
	**KIR2DS3**	**C1**	C*0701	-	-	+	-	-	-	**KIR2DS3**
			C*0304	+	-	-	-	-	-	
			C*0702	-	-	-	-	+	-	
	**KIR2DS4**	**C2**	C*0401	-	+del	-	+del	-	+del	**KIR2DS4**
			C*0501	-	+del	-	+del	-	+del	
	**KIR2DS5**		unknown		-		-		-	**KIR2DS5**
	**KIR3DS1**		unknown		-		-		-	**KIR3DS1**

For NK cells: - and green, NK cells are negative for KIR; + and red, NK cells are positive for KIR; +del and red, NK cells have partial deletion KIR2DS4; + and blue, NK cells have been educated in the context of the cognate HLA. For GBM: +, GBM are positive for the specific HLA ligand; -, GBM are negative for the HLA ligand. Red, GBM are positive for at least one of the HLA ligands of the cognate KIR; green, GBM are negative for all HLA ligands of the cognate KIR; and white, HLA ligand is unknown. **C2 epitope recognized by KIR2DL2/3.

Bortezomib Inhibits Autophagic Flux

Given that oxidative stress, mitochondrial function and turnover are intricately linked to autophagy [2], we investigated whether autophagic flux was affected in P3 GBM cells with the greatest decrease of mitochondria function after NK cell treatment. Autophagy has a dual role in promoting tumor cell survival and death at different stages of disease or treatment [3,4], in a cell specific manner. Damaged proteins induce the biogenesis of double-membraned vesicles known as autophagosomes which sequester cytosolic and damaged proteins, and upon fusion with lysosomes, the contents become degraded [5].

Treatment with BTZ for 24 h modestly increased levels of p62 (sequesstosome-1, SQSTM1) in P3 cells, but not lipidated LC3B (Figure S2A). In contrast, NK cells alone and combination with BTZ reduced both lipidated LC3B-II and p62 expression (Figure S2A) potentially indicating sustained autophagic flux. Flow cytometric analyses of intracellular p62 in P3 and BG7 corroborated the accumulation of p62 after 24 h treatment with BTZ indicated as increased % of cells expressing p62 in P3 (12.5 nM, $p < 0.001$, Figure S2B) and increased mean fluorescence intensity in BG7 cells treated with 6.25 or 12.5 nM BTZ ($p < 0.0001$, Figure S2C). In contrast, combination BTZ + NK cell treated GBM cells reduced the fraction of cells with intracellular p62 for 6.25 nM and 12.5 nM BTZ treated P3 ($p < 0.01$, both) and in 2012-018 and BG7 after 12.5 nM BTZ treatment ($p < 0.05$ and $p < 0.0001$, respectively, Figure S2B), although the mean fluorescence intensity of p62 increased after 6.25 nM (BG7, $p < 0.05$) and 12.5 nM BTZ combination treatment of GBM cells (P3, $p < 0.05$ and BG7, $p < 0.0001$, Figure S2C), consistent with their aggregation as p62-bodies.

Co-staining of the lysosomal compartment with Lysotracker reagent and LC3-positive puncta confirmed negligent levels of LC3 coated lysosomes in untreated control P3 cells, and in NK cell treated GBM cells, findings that are consistent with intact autophagic flux (Figure S2D, left panels). However, lysosomes accumulated in the P3 GBM cells after BTZ or combination BTZ + NK cell treatment (Figure S2D, left panels). Addition of the lysosomal inhibitor chloroquine (CQ) led to markedly accumulated LC3-positive puncta confirming autophagic flux in untreated control as well as after NK cell monotherapy (Figure S2D, right panels).

As further confirmation that BTZ and combination treatment abrogated autophagic flux, we utilized the tetracycline inducible autophagy sensor mCherry-GFP-LC3B to dynamically monitor the formation of autophagosomes vs. autolysomes over time in the presence or absence of CQ (Figure S2E,F). When autophagic flux is intact, fusion of the lysosome with the autophagosome to form autolysosomes occurs, and the acidic pH quenches the GFP fluorescence, causing the autolysomes to appear red under fluorescence microscopy. However, when autophagic flux is blocked, the formed autophagosome puncta are positive for both GFP and mCherry, and thus appear yellow under fluorescence. In untreated controls or after NK cell monotherapy, predominantly red puncta/autolysomes were present indicating sustained autophagic flux (Figure S2E). In contrast, increased yellow puncta/autophagosomes (representing ratio of yellow vs. red puncta) after BTZ alone or in combination with NK cells showed blocked autophagy flux relative to untreated control and NK cell monotherapy (relative to NK cell monotherapy: BTZ 6.25 nM $p < 0.05$, BTZ 12.5 nM $p < 0.001$, BTZ 6.25 nM +NK $p < 0.05$, and BTZ 12.5 nM +NK $p < 0.0001$; relative to control: BTZ 12.5 nM $p < 0.05$ and BTZ 12.5 nM +NK $p < 0.05$, Figure S2E and 2F). Addition of CQ increased the density of yellow autophagosomes, confirming autophagic flux ($p < 0.05$; Figure S2E,F).

Transmission electron microscopy confirmed morphologically the presence of numerous fused, degradative autolysosomes in the cytoplasm of the untreated control P3 cells, as well as after NK cell monotherapy, although P3 mitochondria appeared more electron dense with poorly defined cristae (Figure S2G). Large aggregated, double-membrane limited autophagosomes containing undegraded organelles were apparent in both BTZ (6.25 nM and 12.5 nM) monotherapy and after combination BTZ + NK cell treated P3 cells (Figure S2G) confirming blockade of autophagy flux. Addition of CQ reproduced the large aggregated double-membrane limited autophagosomes containing undegraded organelles. Taken together, these data indicate that BTZ interrupted autophagic flux in P3 and BG7 GBM cells regardless of treatment regimen.

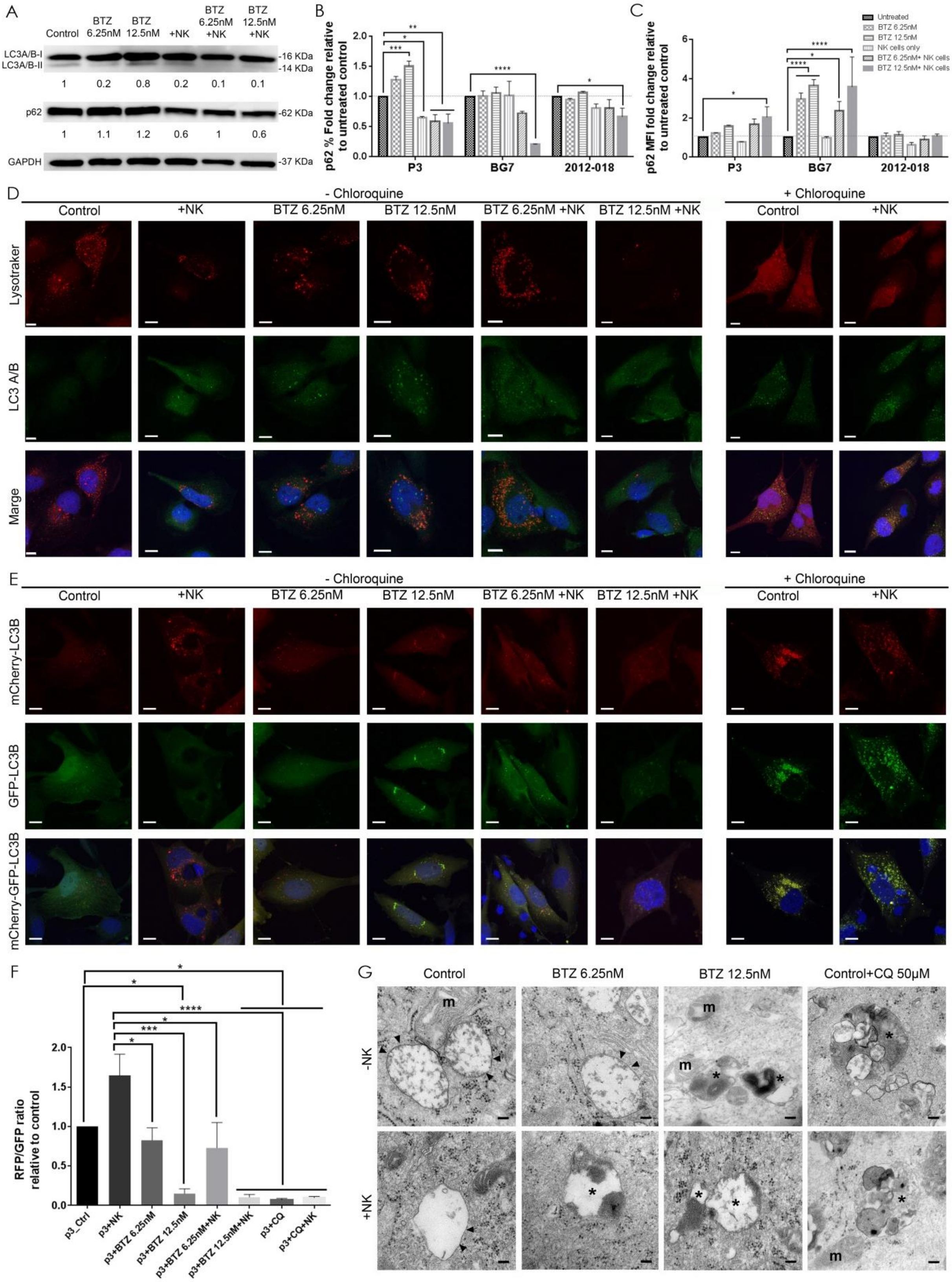

Figure S2. Autophagy characterisation of the different treated GBM cells. (**A**) Western blot analysis of LC3A/B-II and p62(SQSTM1) proteins in lysates from P3 cells untreated and treated with 6.25 and 12.5 nM BTZ, 5:1 NK cells and combination 6.25 nM and 12.5 nM of BTZ + 5:1 NK cells. GAPDH was used as a loading control and for normalizing densitometry measurements of the blots. p62 (**B**) % and (**C**) MFI fold change relative to untreated control of P3, BG7 and 2012-018 GBM cells treated with 6.25 nM BTZ, 12.5 nM BTZ, 5:1

NK cells and combination of 6.25 nM or 12.5 nM of BTZ with 5:1 NK cells. Confocal microscopy of P3 cells before and after treatments stained for (**D**) lysosomal compartment (Lysotraker, red) and LC3 A/B (green) and for (**E**) the Autophagy Tandem Sensor mCherry-GFP-LC3B probe, which gives mCherry-LC3B (red) and GFP-LC3B (green), as a measure of the autophagy flux, (left panel) without chloroquine (CQ) and (right panel) after chloroquine autophagy inhibition. Autophagosome punctae formation (yellow) is indicated in merged and mCherry-GFP-LC3B, respectively, as well as DAPI stained nuclei (blue). (**F**) Quantification of yellow punctae positive cells relative to RFP in untreated controls. (**G**) Transmission electron microscopy of untreated (control), BTZ 6.25 and 12.5 nM as well as control with 50 μM of CQ (upper panels) without NK cell and (lower panels) with 5:1 NK cells. Arrow heads: double-membrane limited autophagosomes; m: mitochondria; *: large aggregated double-membrane limited autophagosomes containing undegraded organelles. Scale bar 100 μm, Magnification 40–100K. Data represents the mean ± SEM of $n = 3$ independent experiments. Two-way ANOVA, Bonferroni's multiple comparison test, * $p < 0.05$; ** $p < 0.01$; *** $p < 0.001$; **** $p < 0.0001$. Western blots in Figures 2F and 3A are from the same experiment on the clipped blot and represent 2 independent experiments.

References

1. Wang, J.; Svendsen, A.; Kmiecik, J.; Immervoll, H.; Skaftnesmo, K.O.; Planagumà, J.; Reed, R.K.; Bjerkvig, R.; Miletic, H.; Enger, P.Ø.; et al. Targeting the NG2/CSPG4 proteoglycan retards tumour growth and angiogenesis in preclinical models of GBM and melanoma. *PLoS ONE* **2011**, *6*, e23062.
2. Galluzzi, L.; Baehrecke, E.H.; Ballabio, A.; Boya, P.; Bravo-San Pedro, J.M.; Cecconi, F.; Choi, A.M.; Chu, C.T.; Codogno, P.; Colombo, M.I.; et al. Molecular definitions of autophagy and related processes. *EMBO J.* **2017**, *36*, 1811–1836.
3. Sui, X.; Chen, R.; Wang, Z.; Huang, Z.; Kong, N.; Zhang, M.; Han, W.; Lou, F.; Yang, J.; Zhang, Q.; et al. Autophagy and chemotherapy resistance: A promising therapeutic target for cancer treatment. *Cell Death Dis.* **2013**, *4*, e838.
4. Kanzawa, T.; Germano, I.M.; Komata, T.; Ito, H.; Kondo, Y.; Kondo, S. Role of autophagy in temozolomide-induced cytotoxicity for malignant glioma cells. *Cell Death Differ.* **2004**, *11*, 448–457.
5. Mizushima, N.; Yoshimori, T.; Ohsumi, Y. The role of Atg proteins in autophagosome formation. *Annu. Rev. Cell Dev. Biol.* **2011**, *27*, 107–132.

© 2019 by the authors. Licensee MDPI, Basel, Switzerland. This article is an open access article distributed under the terms and conditions of the Creative Commons Attribution (CC BY) license (http://creativecommons.org/licenses/by/4.0/).
